# Dihydropyridine-derived calcium channel blocker as a promising anti-hantavirus entry inhibitor

**DOI:** 10.3389/fphar.2022.940178

**Published:** 2022-08-29

**Authors:** Bin Wang, Jiawei Pei, Hui Zhang, Jia Li, Yamei Dang, He Liu, Yuan Wang, Liang Zhang, Libin Qi, Yuewu Yang, Linfeng Cheng, Yangchao Dong, Airong Qian, Zhikai Xu, Yingfeng Lei, Fanglin Zhang, Wei Ye

**Affiliations:** ^1^ Center of Clinical Aerospace Medicine, Airforce Medical University: Fourth Military Medical University, Xi’an, Shaanxi, China; ^2^ Department of Microbiology, School of Preclinical Medicine, Airforce Medical University: Fourth Military Medical University, Xi’an, Shaanxi, China; ^3^ Bone Metabolism Lab, School of Life Sciences, Northwestern Polytechnical University, Xi’an, Shaanxi, China; ^4^ Department of Neurology, Xi’an International Medical Center Hospital, Xi’an, China; ^5^ Student Brigade, School of Preclinical Medicine, Airforce Medical University: Fourth Military Medical University, Xi’an, Shaanxi, China

**Keywords:** Hantaan virus, hantavirus, bunyavirales, hemorrhagic fever with renal syndrome, hantavirus pulmonary syndrome, antivirals, benidipine hydrochloride, calcium channel blocker

## Abstract

Hantaviruses, the causative agent for two types of hemorrhagic fevers, hemorrhagic fever with renal syndrome (HFRS) and hantavirus pulmonary syndrome (HPS), are distributed from Eurasia to America. HFRS and HPS have mortality rates of up to 15% or 45%, respectively. Currently, no certified therapeutic has been licensed to treat hantavirus infection. In this study, we discovered that benidipine hydrochloride, a calcium channel blocker, inhibits the entry of hantaviruses *in vitro*. Moreover, an array of calcium channel inhibitors, such as cilnidipine, felodipine, amlodipine, manidipine, nicardipine, and nisoldipine, exhibit similar antiviral properties. Using pseudotyped vesicular stomatitis viruses harboring the different hantavirus glycoproteins, we demonstrate that benidipine hydrochloride inhibits the infection by both HFRS- and HPS-causing hantaviruses. The results of our study indicate the possibility of repurposing FDA-approved calcium channel blockers for the treatment of hantavirus infection, and they also indicate the need for further research *in vivo*.

## Introduction

Hantaviruses, the causative agents for hemorrhagic fever with renal syndrome (HFRS) and hantavirus pulmonary syndrome (HPS) worldwide (Jiang et al., 2017), belong to the genus *Orthohantavirus*, family *Hantaviridae*, within the order *Bunyavirales*. Hantaviruses are enveloped viruses containing tripartite negative-stranded RNA and a large (L) genome, including medium (M) and a small (S) segment, which encode RNA-dependent RNA polymerase (RdRp or LP), glycoprotein precursor (GPC), and nucleocapsid protein (NP), respectively (Vaheri et al., 2013). During virus maturation, GPC is cleaved into Gn and Gc by the host enzyme, and this cleavage facilitates virus attachment to cellular receptors and subsequent membrane fusion (Cifuentes-Munoz et al., 2014; Mittler et al., 2019; Serris et al., 2020).

The distribution of HFRS and HPS was defined by the geographical distribution of the natural host of these causative viruses (Kabwe et al., 2020). HFRS is mainly endemic in Eurasia, primarily caused by the Hantaan virus (HTNV), Seoul virus (SEOV), Dobrava virus (DOBV), and Puumala virus (PUUV). HPS is an epidemic in the Americas, with SNV and ANDV as the predominant pathogens. HTNV and SEOV are the two primary pathogens of HFRS in China, and HTNV is responsible for severe cases (Jonsson et al., 2010). There are currently no certified pharmaceutical agents approved by the U.S. Food and Drug Administration (FDA) to treat hantavirus infection, considering the mortality of up to 15% for HFRS and 45% for HPS, respectively. It underlines the urgent need to develop new therapeutic antivirals (Brocato and Hooper, 2019; Ye et al., 2019; Munir et al., 2020). The repurposed use of FDA-approved drugs is an effective strategy to identify potential antivirals, considering clinical safety, and was adopted during the SARS-CoV-2 pandemic (Riva et al., 2020; Santos et al., 2020; Zhou et al., 2020). In addition to SARS-CoV-2, this strategy has been applied to various emerging and re-emerging viruses, such as the Ebola virus, Zika virus, and severe fever with thrombocytopenia syndrome virus (SFTSV) (Johansen et al., 2015; Barrows et al., 2016; Li et al., 2019).

Hantavirus, an enveloped virus, binds to cellular receptors through viral glycoprotein and enters cells *via* endocytosis (Mittler et al., 2019; Noack et al., 2020; Guardado-Calvo and Rey, 2021). The interfering entry process is, therefore, an attractive strategy for combating hantavirus infection. Vesicular stomatitis virus (VSV) is widely used to screen viral entry inhibitors due to its multiple advantages including the ability to be handled under lower biosafety conditions and the ability to pseudotype various viral envelope proteins (Mayor et al., 2021). Based on recombinant VSV-based antiviral screening, we found that benidipine hydrochloride, a calcium channel inhibitor, is a potential beneficial countermeasure against hantavirus. By blocking membrane-bound calcium channels, calcium channel inhibitors reduce the intracellular calcium level, which may have protective effects on vascular endothelial cells (Yao et al., 2006). Furthermore, benidipine HCl exhibits pan-anti-hantaviral activity when applied to pseudotyped viruses carrying different hantaviral glycoproteins. As a result of our study, we believe that benidipine HCl and other calcium channel inhibitors hold promise as potential therapeutic agents for treating hantavirus infection. We believe further *in vivo* studies are warranted.

## Methods

### Cells, viruses, and reagents

African green monkey kidney Vero-E6 (ATCC, CCL-81), human non-small-cell lung carcinoma (A549) (ATCC; CCL-185), Syrian golden hamster kidney BHK-21 (ATCC; CCL-10), human embryonic kidney HEK-293T, and human hepatoma Huh7 cells were cultivated in Dulbecco’s modified Eagle’s medium (DMEM, Sigma-Aldrich, St. Louis, MO, United States) supplemented with 10% fetal bovine serum (FBS, Sigma-Aldrich) in 5% CO_2_ at 37°C, as described previously (Ye et al., 2020). DMEM without Ca^2^^+^ was obtained from Yuchun Bio (Shanghai, China).

HTNV (strain 76-118) was propagated and titrated in Vero-E6 cells, as previously indicated (Ye et al., 2015; Ye et al., 2019).

The primary mouse monoclonal antibodies against HTNV NP (1A8) were produced in a lab, as mentioned earlier (Cheng et al., 2016). An antibody against GFP was purchased from Abbkine (Wuhan, China), and tubulin/GAPDH was purchased from Sangon Biotech (Shanghai, China). Horseradish peroxidase (HRP) or infrared dye-conjugated secondary antibodies were obtained from Sangon Biotech and Li-Cor Biosciences (Lincoln, NE, United States), respectively.

Benidipine hydrochloride (HCl) was purchased from MedChemExpress (NJ, United States) and cilnidipine, felodipine, nicardipine HCl, nifedipine, nisoldipine, and nitrendipine were purchased from TargetMol (Shanghai, China). Manidipine was purchased from APExBIO Technology (Houston, TA, United States). The calcium chelator BAPTA-AM was purchased from GlpBio (Montclair, CA, United States).

### Cytotoxicity assay

Cell viability was calculated as previously mentioned (Ye et al., 2020). Briefly, Vero-E6 and A549 cells were incubated with serial dilutions of each drug for 24–48 h. Cell viability was assayed using Cell Counting Kit-8 (CCK8) (TargetMol), with absorbance (A) at 450 nm being measured using a BioTek HT synergy instrument.

### Rescue of recombinant vesicular stomatitis virus bearing the Hantaan virus glycoprotein precursor

The plasmid-bearing VSV antigenome lacking VSV-G ORF but with an additional GFP ORF was synthesized at GenScript (Nanjing, China), containing unique restriction sites for foreign gene expression: 5′ flanked by the T7 bacteriophage promoter, 3′ flanked by hepatitis delta virus ribozyme (HDVRz), and the T7 terminator sequence. The codon-optimized GPC genes of HTNV (NC_005219) were PCR amplified and inserted into the VSV antigenome plasmid, resulting in the rVSV-HTNV-G vector.

BHK-21 cells were seeded into a six-well plate overnight and infected with vaccinia virus bearing T7-pol (kindly provided by the Wuhan Institute of Virology, CAS) for 2 h. Then, the cells were transfected with helper plasmids encoding VSV-N, VSV-P, VSV-L, and VSV-G (kindly provided by the Wuhan Institute of Virology, CAS). The transfection ratio for each plasmid was 5:3:5:1:8, with a total of 11 μg per well. Transfections were performed using the Hieff Trans Liposomal Transfection Reagent (Yeasen, Shanghai, China). Cytarabine (TargetMol) was added after transfection at a concentration of 100 μg/ml, and the culture supernatant was collected 72 h post-transfection and used to blind infect VeroE6 cells. After several passages, the cytopathic effect (CPE) in the cell monolayer was noticeable and indicated a successful rescue. The rescued virus was referred to as rVSV-HTNV-G and verified by HTNV GPC-specific antibodies. Viruses were propagated and tittered on Vero E6 cells, and the titer was determined using plaque assays.

### Preparation of the hantavirus glycoprotein precursor pseudotyped vesicular stomatitis virus

GPC genes of HTNV (NC_005219), SEOV (AB027521), PUUV (U14136), DOBV (L33685), ANDV (AF291703), and SNV (L25783) were codon-optimized and synthesized at GenScript (Nanjing, China). All target genes were cloned into the pCAGGS vector, as previously indicated (Yao et al., 2020). Pseudotyped VSV (pVSV∆G-GFP) was stored in our laboratory, where the coding region of the G protein was replaced with an enhanced green fluorescent protein, and the VSV-G protein was expressed in the trans form. BHK-21 cells were transfected with pCAGGS-GPC of each hantavirus, 24 h later, and then pVSV∆G-GFP-bearing VSV-G was added at a multiplicity of infection (MOI) of 1 for 1 h at 37°C. The monolayer was then washed with Dulbecco’s Phosphate-Buffered Saline (DPBS, Cellgro) three times, and the fresh medium was replenished. After 36 h of incubation, the culture supernatant was clarified by low-speed centrifugation, aliquoted, and stored at −80°C.

### Western blot analysis

As indicated in different experiments, cells in six-well plates were treated with benidipine HCl and infected with viruses. Cells were washed twice with DPBS and lysed with RIPA buffer (Beyotime, P0013C, or P0013D). Samples were quantified using a BCA kit (Thermo Fisher Scientific), and 20 μg or 40 μg aliquots of each cell lysate were boiled for 10 min and subjected to 12% SDS-PAGE and then transferred to polyvinylidene difluoride (PVDF) membranes (Millipore) and blotted with indicated primary antibodies, followed by secondary antibodies conjugated to infrared dyes or HRP and visualized using an Odyssey Infrared Imaging System (Li-Cor Biosciences) or Tanon 5200SF Imaging System (Shanghai, China).

### Immunofluorescence assay

Cells in 24-well plates were treated with drugs and infected with rVSV, rVSV-HTNV-G, or different pVSV and were imaged with an IX71 fluorescence microscope (Olympus, Tokyo, Japan) 24 h post-infection. For the immunofluorescence assay, cells were seeded onto coverslips in 24-well plates at a confluence of 60%–70%. Then, cells were treated with benidipine HCl and infected with HTNV, subjected to IFA at the indicated time points, p. i., following an established protocol (Ye et al., 2019). Cells were imaged with a BX60 fluorescence microscope (Olympus).

### Quantitative reverse transcription PCR

Total RNA of HTNV or rVSV-HTNV-G infected cells was extracted and reverse transcripted using the Hifair® 1st Strand cDNA Synthesis SuperMix (Yeasen), according to the instructions provided by the manufacturer. qRT-PCR was performed using the Hieff® qPCR SYBR Green Master Mix (Yeasen) on a CFX96 Real-Time system (Bio-Rad). The mRNA expression level of each target gene was normalized to the corresponding GAPDH expression level. The primers used for gene amplification were as follows: GFP (forward: 5′-CTG​GAC​GGC​GAC​GTA​AAC​G -3’; reverse: 5′-CCA​GGG​CAC​GGG​CAG​CTT​GC -3′), HTNV S segment (forward: 5′-GAG​CCT​GGA​GAC​CAT​CTG -3’; reverse: 5′-CGG​GAC​GAC​AAA​GGA​TGT -3′), and GAPDH (forward: 5′- ACC​CAC​TCC​TCC​ACC​TTT​G -3’; reverse: 5′- ATC​TTG​TGC​TCT​TGC​TGG​G -3′).

### Statistical analysis

Statistical analysis was performed using a two-tailed unpaired t-test in GraphPad Prism software (La Jolla, CA, United States). Data are presented as means ± standard deviations (SDs) (*n* = 3 or otherwise indicated). All experiments were repeated at least three times.

## Results

### Benidipine hydrochloride inhibits Hantaan virus infection in different cell lines

**FIGURE 1:**
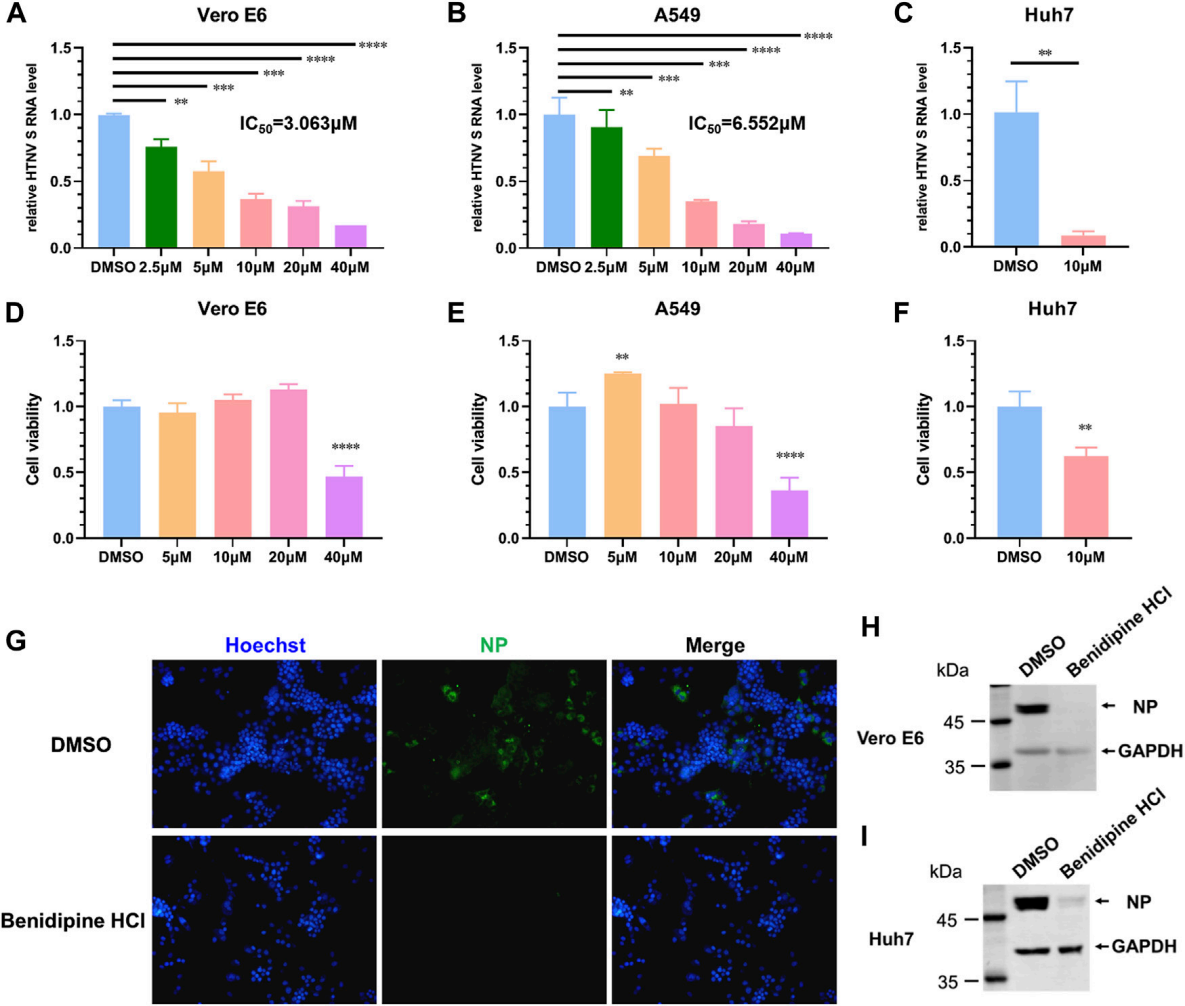
Benidipine hydrochloride inhibits HTNV infection. **(A–C)** Benidipine hydrochloride inhibited HTNV in Vero, A549, and Huh7 cells with dose-dependent effects. Benidipine hydrochloride was applied to cell monolayers at the indicated concentrations for 1 hour, and then, HTNV was inoculated at MOI 1 with the same concentrations of benidipine hydrochloride. After adsorption, the inoculum was discarded, and benidipine hydrochloride was added to the fresh medium. Total RNA was extracted 24 hpi, and the HTNV S segment RNA level was measured by qRT-PCR and normalized to GAPDH. IC_50_ was indicated in the panel (IC_50_ refers to 50% inhibitory concentration). Vero cells **(D)**, A549 cells **(E)**, and Huh7 cells **(F)** were analyzed for viability using the CCK8 assay after 24 h of benidipine hydrochloride treatment. The inhibition of the expression of HTNV structural proteins by benidipine hydrochloride. **(G–I)** Vero cells were treated with 10 μM benidipine hydrochloride and infected with HTNV; after 24 h of infection, coverslips were stained with the NP-specific antibody 1A8 **(G)**. After being exposed to 10 μM benidipine hydrochloride, Vero cells **(H)** or Huh7 cells **(I)** were infected with HTNV. After 24 h of infection, cells were lysed and immunoblotted with the antibody 1A8. Student’s t-test was used to compare mean values between the benidipine hydrochloride-treated group and the vehicle control group (DMSO). *p < 0.05; **p < 0.01; ***p < 0.001; ****p < 0.0001.

The repurposing of FDA-approved drugs is an effective method of screening potential antiviral agents since the safety of these drugs has been demonstrated in clinical trials. By leveraging this strategy, we found that benidipine hydrochloride (HCl) significantly inhibited HTNV replication. Benidipine HCl was added to Vero cells 1 hour prior to HTNV infection; the cells were inoculated with HTNV at a multiplicity of infection (MOI) of 1, and benidipine HCl was added during and after virus adsorption. The relative intracellular RNA levels of the HTNV S segment were determined by quantitative real-time PCR (qRT-PCR) at 24 h after infection. A dose-dependent reduction of the HTNV S segment was observed in cell monolayers treated with benidipine in comparison with the DMSO vehicle, with an IC_50_ of 3.063 μM (Figure 1A). However, CCK8 assays did not reveal any effect on cell viability (Figure 1D). Inhibition of HTNV infection by benidipine HCl was also observed on A459 cells with an IC_50_ of 6.552 μM (Figures 1B,E) and Huh7 cells (Figures 1C,F), both permissive cell lines for replication of HTNV. Furthermore, in HTNV-infected Vero E6 cells, treated with benidipine HCl, the number of NP-positive cells was remarkably reduced (Figure 1G). Additionally, benidipine HCl treatment significantly reduced the relative protein levels of NP (Figures 1H,I).

### Benidipine hydrochloride inhibits Hantaan virus infection by blocking virus entry interfering with virus internalization

**FIGURE 2:**
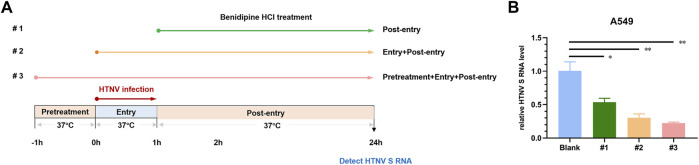
*In vitro* anti-HTNV mechanism of benidipine hydrochloride. **(A)** Benidipine hydrochloride was administered to Vero E6 cells, as described in the schematic diagram. After HTNV infection, cells were treated with benidipine hydrochloride (#1), or added with HTNV prior to adsorption (#2), or 1 h before HTNV and added throughout the infection procedure (#3). **(B)**The effect of benidipine hydrochloride on the entry of HTNV. As depicted in **(A)**, the relative intracellular S segment RNA level of HTNV was determined at 24 h post-infection using a variety of methods. *p < 0.05; **p < 0.01.

Following this, we examined the mechanisms by which benidipine HCl inhibits HTNV infection. Upon treating Vero E6 cells with 10 μM benidipine HCl before virus adsorption, or during virus adsorption and afterward, or after virus adsorption, intracellular levels of the viral S segment RNA were detected 24 h post-infection (Figure 2A). A gradual decrease in the HTNV RNA level was observed over the course of benidipine HCl treatment, during and after the treatment, and after adsorption, suggesting that benidipine HCl inhibited HTNV entry into the cell (Figure 2B).

**FIGURE 3:**
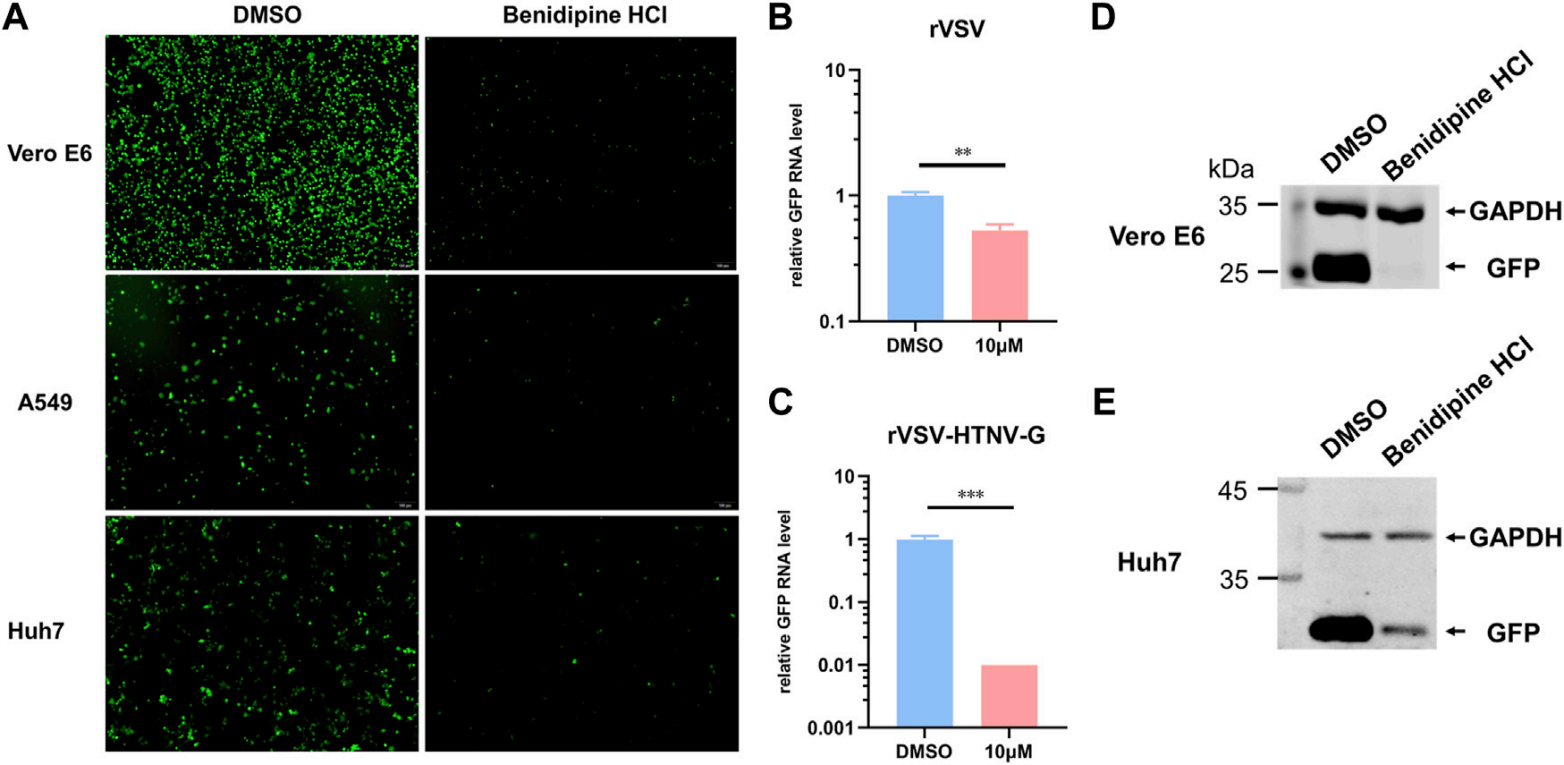
Effect of benidipine hydrochloride on the entry stages of HTNV. **(A)** Vero E6, A549, and Huh7 cells were treated with benidipine hydrochloride or the DMSO vehicle and then inoculated with rVSV-HTNV-G at MOI 1, and GFP-positive cells were photographed 24 h after infection. **(B,C)** Vero E6 cells were treated with benidipine hydrochloride or the DMSO vehicle and then infected with rVSV **(B)** or rVSV-HTNV-G **(C)**. After 24 h post-infection, total RNA was extracted, and GFP RNA levels were determined by qRT-PCR and normalized to GAPDH. **(D, E)** Vero **(D)** and Huh7 **(E)** cells were treated with 10 μM benidipine hydrochloride and infected with rVSV-HTNV-G; the cells were lysed 24 h after infection and blotted with antibodies against GFP and GAPDH. **p < 0.01; ***p < 0.001.

For further validation of benidipine HCl’s entry inhibitory effects on HTNV, we tested its effect on a recombinant VSV expressing GFP with glycoprotein substituted for HTNV GPC (rVSV-HTNV-G). In Figure 3A, the number of GFP-positive cells was significantly lower in benidipine HCl-treated cells than in DMSO-treated cells, indicating that benidipine HCl inhibited rVSV-HTNV-G infection in different cell lines. Additionally, the level of GFP RNA within cells was detected 24 h following infection with rVSV and rVSV-HTNV-G, indicating that benidipine HCl is more effective at inhibiting rVSV-HTNV-G than rVSV (Figures 3B,C). In addition, Vero E6 and Huh7 cells treated with benidipine HCl had significantly lower levels of GFP protein than Vero E6 and Huh7 cells treated with DMSO (Figures 3D,E).

### Calcium channel blockers inhibit Hantaan virus infection through reducing cellular Ca^2+^ uptake

**FIGURE 4:**
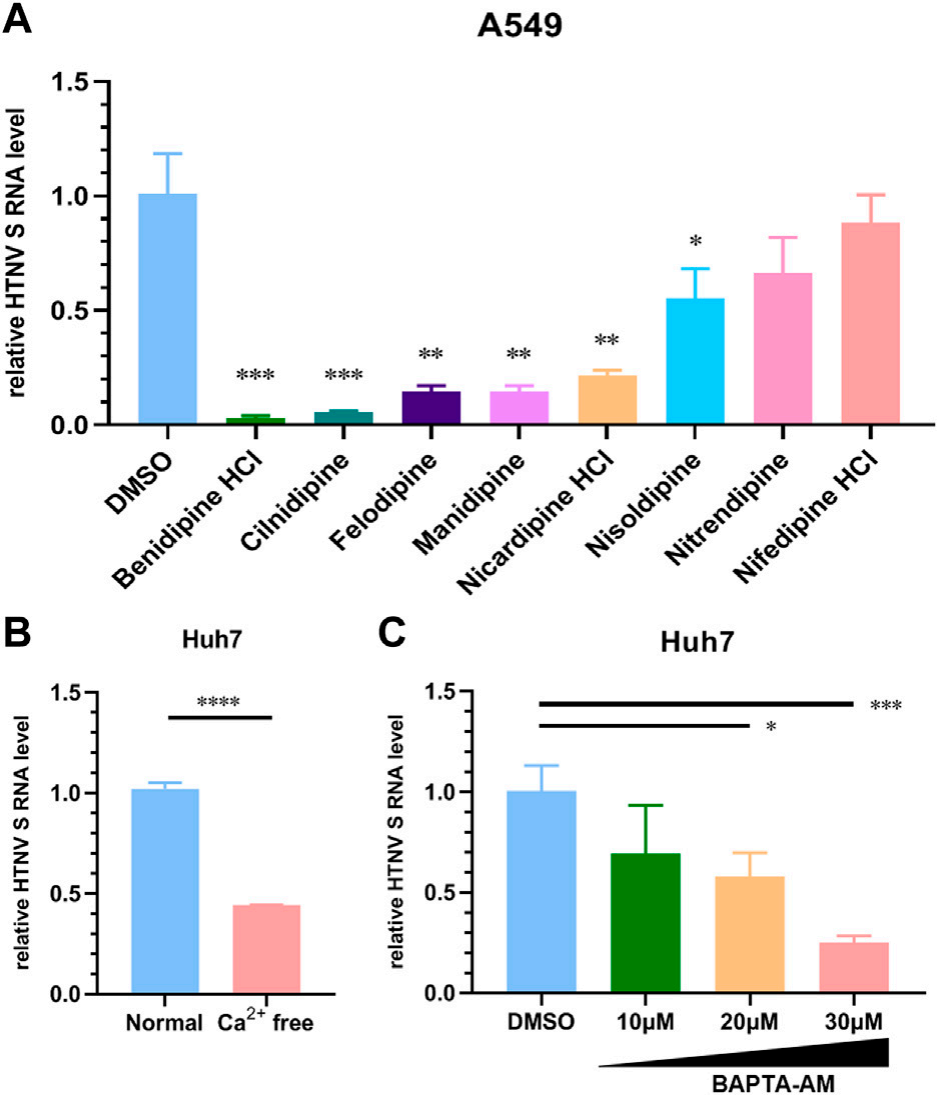
Different dihydropyridine-derived calcium channel blockers inhibit HTNV infection. **(A)** A549 cells were treated with the indicated CCBs or the DMSO vehicle and infected with HTNV at MOI 1, and the relative level of S segment RNA was determined by normalizing to GAPDH. **(B)** Huh7 cells were cultured in Ca^2+^-free or normal medium and then infected with HTNV (MOI = 1), 24 hpi; the relative intracellular HTNV RNA level was measured by qRT-PCR. **(C)** Huh7 cells were infected with HTNV upon treatment with BAPTA-AM, 24 hpi. The relative intracellular HTNV RNA level was measured by qRT-PCR. *p < 0.05; **p < 0.01; ***p < 0.001.

As a calcium channel blocker derived from dihydropyridine (DHP), benidipine HCl is commonly used in the treatment of hypertension. In order to determine whether other members of the DHP process exhibit similar HTNV inhibition functions, we tested the anti-HTNV activity of an array of DHPs. In Figure 4A, cilnidipine, felodipine, amlodipine, manidipine, nicardipine, and nisoldipine inhibit HTNV replication on A549 cells at 10 μM, suggesting that multiple DHP-derived calcium channel blockers have a common anti-HTNV effect. The HTNV RNA level was also significantly reduced when Huh7 cells were treated with Ca^2+^-free medium as compared to the normal medium (Figure 4B). Likewise, the HTNV RNA level was also decreased in a dose-dependent manner when Huh7 cells were treated with BAPTA-AM, a calcium chelator (Figure 4C). Thus, these results suggest that calcium channel blockers inhibit HTNV infection by reducing the Ca^2+^ influx.

### Benidipine hydrochloride exhibits the broad-spectrum anti-hantaviral entry activity

**FIGURE 5:**
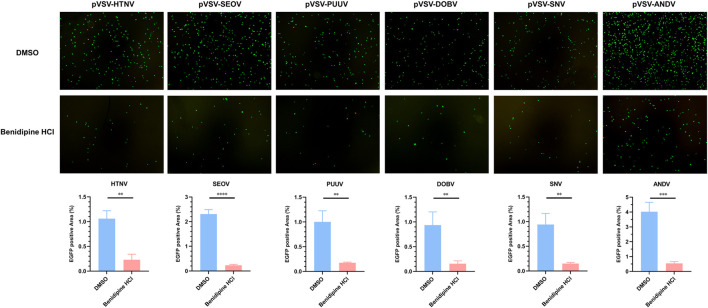
Benidipine hydrochloride inhibits multiple hantaviruses’ glycoprotein pseudotyped VSV infection. Various pseudotyped VSV bearing different hantaviral glycoproteins were incubated with 10 μM benidipine HCl or the vehicle DMSO, after which the mixture was used to infect A549 cells, and the GFP-positive cells were photographed 24 h post-infection. **p < 0.01; ***p < 0.001; ****p < 0.0001.

In order to examine whether benidipine HCl inhibits other hantaviruses, we rescued various VSV pseudoviruses enveloped with pathogenic hantaviral glycoproteins, including SEOV, PUUV, DOBV, SNV, and ANDV. As expected, benidipine HCl significantly decreased the infection rate of those pVSVs (Figure 5), demonstrating that it has a broad-spectrum anti-hantavirus activity.

## Discussion

The development of a drug is a complex process that involves a great deal of uncertainty and takes a long time. The repurposing of licensed drugs is an alternative strategy for the development of antivirals (Johansen et al., 2015; Barrows et al., 2016; Li et al., 2019; Riva et al., 2020; Santos et al., 2020; Zhou et al., 2020). Dihydropyridine-derived calcium channel blockers have been used for a long time to treat hypertension and angina pectoris (Yamamoto et al., 1990). As a new long-acting drug, benidipine HCl is approved for its ability to bind to the voltage-gated calcium channel’s DHP-binding site (Yao et al., 2006). In addition to its anti-hypertensive and cardioprotective properties, benidipine HCl also exhibits renoprotective and endothelial protective properties (Karasawa and Kubo, 1990; Yao et al., 2000; Matsubara and Hasegawa, 2005). In addition, because benidipine HCl only has mild side effects and has a high level of clinical safety, it is attractive for its potential application against other diseases.

As an acute disease with high mortality, HFRS is transmitted primarily through the inhalation or consumption of rodent-contaminated air or food (Jiang et al., 2017; Kabwe et al., 2020). Endothelial permeability increases and renal damage are the main symptoms of HFRS caused by HTNV or SEOV (Jiang et al., 2017). Currently, there are no licensed antivirals against these viruses, and the first-line treatment option is supportive therapy. Since the responsible pathogens are biosecurity-related, treatment measures are needed for these viruses.

The present study demonstrated that benidipine HCl has a powerful antiviral effect on HTNV in a variety of cells with an IC_50_ at a low micromolar level. Furthermore, benidipine HCl significantly inhibited the entry of rVSV-bearing HTNV GPC into cells, and the inhibition is more potent than that of VSV itself. Pretreatment of cells with benidipine HCl during virus infection and after virus adsorption results in the most effective inhibition of HTNV compared to other methods. The results indicate that benidipine HCl inhibits HTNV mainly at the entry stage.

Additionally, both the calcium-free medium and BAPTA-AM, a cellular calcium chelator, inhibit HTNV replication, along with numerous calcium channel blockers derived from DHP. These results suggest that the level of intracellular Ca^2+^ correlates with the replication level of HTNV and other hantaviruses. In fact, West Nile virus (WNV) is one example of a virus that induces Ca^2+^ influx, which is crucial for efficient viral replication (Scherbik and Brinton, 2010). Aside from this, Ca^2+^ can regulate multiple pathways through signal transduction (Bagur and Hajnóczky, 2017) and could serve as an active center for some special proteins, such as the annexin family, which has been shown to be involved in the regulation of multiple virus infections. Furthermore, DHP-derived calcium channel blockers target different calcium channel subtypes and differ in half-life times, which may result in different inhibitory efficacies against HTNV. Nevertheless, further research is necessary to identify the specific mechanism.

Viral hemorrhagic fever (VHF) poses a serious health threat to humans, particularly in less-developed countries. There are several types of viruses that can cause VHF, such as filoviruses, arenaviruses, bunyaviruses, and alphaviruses. Other CCBs, in addition to benidipine HCl, may inhibit VHF-induced virus infection by interfering with the virus entry stage (Lavanya et al., 2013; Sakurai et al., 2015; DeWald et al., 2018; Li et al., 2019). A number of CCBs were also evaluated to see if they were effective in stopping HTNV infection, and multiple drugs were found to be effective. In light of the wide-spectrum nature of CCBs against VHF viruses, we investigated the effect of benidipine HCl against hantavirus pseudotyped viruses and concluded that this agent was able to inhibit the main pathogenic hantaviruses responsible for both HFRS and HPS. This host-targeting treatment is unlikely to result in drug-resistant strains. Further research into how Ca^2+^ regulates the replication of HTNV may provide additional information regarding the hantavirus lifecycle and strengthen our understanding of its virology.

Overall, we found that benidipine HCl, as well as other CCBs, can inhibit hantavirus infection. This is similar to what has been observed for other viruses, which indicates that an *in vivo* study of benidipine HCl against hantavirus infection is warranted.

## Data Availability

The original contributions presented in the study are included in the article; further inquiries can be directed to the corresponding authors.
